# Editorial: Physiology in Extreme Conditions: Adaptations and Unexpected Reactions

**DOI:** 10.3389/fphys.2017.00748

**Published:** 2017-09-29

**Authors:** Maria G. Trivella, Enrico Capobianco, Antonio L'Abbate

**Affiliations:** ^1^Consiglio Nazionale delle Ricerche, Istituto di Fisiologia Clinica, Pisa, Italy; ^2^Center for Computational Science, University of Miami, Miami, FL, United States; ^3^Scuola Superiore Sant'Anna, Pisa, Italy

**Keywords:** extreme environments, adaptation, homeostasis, space, underwater, sports

## Outreach

“Physiology in extreme conditions” was concerned with two main issues:

(a) to increase knowledge about individual reactions and mechanisms of adaptations in specific extreme environmental conditions;

(b) to widen the analytical skills gap created by data generated in extremely severe conditions, and in response to pathological disorders.

A wide spectrum of topics was proposed by the included articles, which covered a wide variety of cases ranging from cellular systems to preclinical models. The focus was on healthy individuals experiencing extreme conditions induced by typically non-physiological environments. Studies of pathological relevance encompassing artificial organs were also comprised in the Research Topic.

## Lessons learned

While many mechanisms could simply be associated with adaptations as evidenced by studies in sport, other mechanisms would rather refer to unexpected reactions in response to internal stimuli and/or external abrupt changes, as showed by the evaluation of psychostimulant abusers and/or unmedicated psychiatric patients.

In particular, the similarity noticeable in the behavioral symptoms shared by extremely agitated drug addicts and untreated psychiatric patients has been linked to a possible genetic disorder leading to a dysregulated central dopamine transporter function. This is the case with the biologically precipitating cause of acute delirium and sudden death (Mash).

The end-stage heart failure represents a condition where the cardiovascular system fails to maintain the blood supply to all organs and tissues and is generally a result of chronic disease in adults or of cardiac congenital abnormalities in children. This is a very serious condition with no optimal therapeutic treatments that can ultimately improve the situation to a point where mortality is avoided.

The development of durable ventricular assist devices (VADs) has reduced mortality rates and quality of life in patients with this critical condition. The mechanical support in smaller children is an effective strategy for bridging patients to heart transplantation (Di Molfetta et al.). The option of total artificial heart has been approved for humanitarian reasons both in pediatric surgery (Villa and Morales) and in adults (Cohn et al., 2015).

Note that an artificial pump instead of the native heart could be considered as an abrupt interruption of the cardiovascular system continuity (endothelial lining, cell to cell communication signaling, neuro-humoral, and neuro-hormonal molecules production, sympathetic-parasympathetic balance as well as central perception of the substituted organ, i.e., not a simple pump). It is crucial to stress that the findings of cardiac support by VADs and artificial heart could clarify the physiological and the physio-pathological mechanisms of the cardiovascular system.

## Specific added value of studies performed in extreme conditions

We considered whether physiology in extreme conditions can reveal important reactions of the human body and can help the process of assessing the limits emerging under healthy conditions and critical signals of transition toward disease. Underwater environment is one in which many body functions are critically stressed (Pendergast and Lundgren, 2009).

Relevant answers were for instance found in the study on the effect of pre-dive whole body vibration on post-dive bubble formation: Balestra's paper referred that the whole body vibration with the diver laying on a vibrating mattress was much a better condition than preconditioning with oxygen for a reduction of decompression-induced vascular gas emboli (Balestra et al.). Evidence that intensive weight reduction regimens induce changes in female body composition and serum hormones was demonstrated by Hulmi et al.'s study, in which it was observed recovery in subsequent months owing to increased energy intake.

Space is another type of special environment that requires extreme changes and adaptations for living organisms (plants, animals, and human beings). Space flight imposes constraints on the body of highly selected, well-trained and healthy subjects (Aubert et al., 2016; Bergouignan et al., 2016). In particular, the pathophysiological adaptive changes resemble an accelerated aging process and relate with some disease processes (Fitts et al., 2000; Vernikos and Schneider, 2010). As such effects become manifest over a time span of weeks (i.e., cardiovascular deconditioning) to months (i.e., loss of bone density and muscle atrophy) of exposure to weightlessness, some kinds of corrections are possible during flight. Also note that, in due time, these effects are mostly reversible after landing (Demontis et al.).

Overall, a variety of stressors has been widely analyzed in preclinical models and in human studies. What is clear is that a pathway underlying such conditions is driven by inflammation, and this explains the multiple effects visible through multiple associated morbidities.

## Translational relevance

In the translational field, with reference to the study on the fluoxetine protection in decompression sickness in mice crosslinks molecular biology, pharmacological actions, and basic mechanisms of diseases like disseminated coagulation, inflammation, and ischemia induce neurological damage and death. These symptoms result from circulating bubbles generated by a pathogenic decompression: acute fluoxetine treatment increases the survival rate of mice subjected to an experimental dive, enhanced by TREK-1 inhibition (Vallée et al.).

Another adaptation in extreme acidic environment is analyzed by the study on hydrothermal vent organisms, which have evolved physiological adaptations to cope with extreme abiotic conditions including temperature and pH (Hu et al.). The considerable acid-base regulatory abilities in brachyuran crabs could suggest some basic mechanisms in support of both the understanding of the derangements in acid-base homeostasis and the design of mock systems addressing environmental safety.

We notice that the observations derived from animal behavior in terrestrial and marine environment are currently used for bioengineering studies and innovative research. Bio-robotic investigational areas could be linked to physiology in extreme conditions for foresight programs.

## Ethical aspects

By considering the ethical request of animal experiments reduction, an example of alternative methods is offered by the study of hepatic cells, prospecting a genetic therapeutic approach for prophylaxis and treatment of liver fibrosis by a double-knockdown interfering with the intracellular oxygen sensor-prolyl hydroxylase 1 (PHD1) and the intracellular oxidative stress sensor-kelch-like ECH associated protein 1 (Keap1). The findings pointed to increased cell viability and a down-regulated expression of pro-fibrogenic molecules (Liu et al.).

## Sport

A number of studies have been included in the research topic that have used sport as an example of extreme physiological processes. The recent advent of a widespread proliferation of ultra-long endurance races has consequently motivated the evaluation of factors such as the physiological response of the athletes, the cognitive and neurosensory pattern, the sleep deprivation effects (Tonacci et al.). These are all important elements to be considered for safety reasons.

Different types of activities can induce acute and chronic changes with or without recovery of homeostasis afterwards (Ujka et al.): monitoring during and after races physiological variables of different subjects seems to be essential in terms of human health.

Similarly to safety in competitions, and focusing at training, the analysis on the kinematics of the jump appears relevant in order to evaluate the adaptation of the motor cerebral programming to the jumper's physical characteristics, the control of the initial posture, and the jumper's perception of the position of the body mass center, as underlined by some authors (Fargier et al.).

## Physiology challenges, people engagement, and big data support

Biomedical data have increased dramatically in both volume and variety with the emergence of people-integrated health data generating sources, typically referred as m-healh. Additional complexity is expected when tasks such as profiling or risk assessment are required. This calls for analytical improvements, not currently available from specialized rather than integrated disciplines, which in turn calls for a new generation of scientists and clinicians.

*Conditio sine qua non* remains the enhancement of communication skills, shared decision making and definition of disease reclassifications, but also a re-assessment of people engagement in the N-of-1 or individualized medicine era. While the Big Data realm of applications promises to be relevant and rich with opportunity, but benefits to people will come with harmonization among all specialized individuals operating with data (Beckmann and Lew, 2016).

## Concluding remarks

Other conditions beyond those presented in this research topic deserve future attention: namely, altitude (Grocott et al., 2007), consciousness abolished in anesthesia, sleep deprivation, and circadian rhythms, breakdown in sudden events, and catastrophes. Thinking ahead, all such studies belong to a novel, interdisciplinary and highly complex research field, which nevertheless could stimulate the curiosity of researchers and clinicians, hopefully inducing further interactions within joint scientific activities.

## Author contributions

All authors listed have made a substantial, direct and intellectual contribution to the work, and approved it for publication.

### Conflict of interest statement

The authors declare that the research was conducted in the absence of any commercial or financial relationships that could be construed as a potential conflict of interest.
